# The complete plastid genome of the brown alga *Scytosiphon lomentaria* (scytosiphonaceae, phaeophyceae)

**DOI:** 10.1080/23802359.2019.1623726

**Published:** 2019-07-10

**Authors:** Kuipeng Xu, Bin Zhou, Yan Sun, Yu Zang

**Affiliations:** College of Marine Life Sciences, Ocean University of China, Qingdao, China

**Keywords:** *Scytosiphon lomentaria*, plastid genome, phylogenetic analysis

## Abstract

The complete plastid genome of *Scytosiphon lomentaria* was determined in this study. The circular genome was 139,208 bp in length with the GC content of 31.3%. It contained 137 protein-coding genes (PCGs), 27 transfer RNA (tRNA) genes, and 8 ribosome RNA (rRNA) genes. A phylogenetic analysis based on the plastid genomes of Phaeophyceae indicated that *S. lomentaria* is located in the Ectocarpales lineage, being closely related to *Endarachne binghamiae.*

*Scytosiphon lomentaria* is believed to include some cryptic species, which is a widely distributed in cold and warm waters worldwide (Kogame et al. 2015). This species belongs to the family Phaeophyceae of the order Ectocarpales and grows attached to shells and stones in intertidal or shallow subtidal zones. Previous molecular phylogenetic studies have shown that *S. lomentaria* appeared to considerable genetic diversity (Yuon Cho et al. 2007). At present, there has been no plastid genome studies on *S. lomentaria*. We aimed to assemble and characterize *S. lomentaria* genome to provide a better understanding on the evolution and genetics of Phaeophyceae and other species in the same family.

In this study, the complete plastid genome of *S. lomentaria* was initially assembled from previously published Illumina sequencing data (SRR5026350), which was used as a PCR primer guide. This specimen was collected from the beach of Xinghai Bay, China. The specimen and DNA were stored in Gene Denovo Laboratory. The whole mitochondrial genome was sequenced with 140 pairs of primers and assembled using CAP3 software (Huang and Madan 1999). The assembled plastid genome was then annotated by DOGMA (Wyman et al. 2004) and was submitted to GenBank with accession numbers MK798154. The complete *S. lomentaria* plstid genome is 134,485 bp in length and contained a pair of inverted repeat regions (5544 bp each), a 80,468 bp large single copy (LSC) region, and a 42,929 bp small single copy (SSC) region. The circular genome encoded a total of 172 genes, consisting of 137 protein-coding genes (PCGs), 27 transfer RNA (tRNA) genes, and 8 ribosome RNA (rRNA) genes. The overall GC content is 31.3%.

A phylogenetic analysis was carried out with *S. lomentaria* and 10 other complete plastid genomes of species from the Stramenopiles group. *Porphyra yezoensis* from the Rhodophyta was included as outgroup. 86 concatenated protein-coding amino acid sequences were aligned using the program MAFFT (Katoh et al. 2005) and were trimmed using trimAl with the option ‘automated1’ (Capella-Gutiérrez et al. 2009). Maximum likelihood (ML) analysis was conducted using RaxML with the cpREV + G+I substitution model (Stamatakis 2014). The position of *S. lomentaria* in the clade of Phaeophyceae was highly supported (Figure 1). The ML tree indicated that *S. lomentaria* locating in the Ectocarpales lineage, being closely related to *E. binghamiae*.

**Figure 1.**
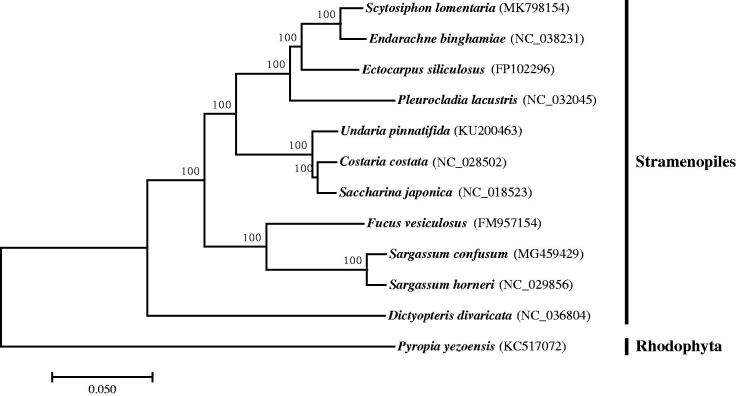
Maximum likelihood (ML) phylogenetic tree of the *Scytosiphon lomentaria* and 11 other species based on the concatenated sequences of 86 protein-coding genes. Numbers on nodes indicate bootstrap support value, based on 1000 replicates the Genbank accession numbers were in brackets.
